# Obituary

**Published:** 1870-10

**Authors:** 


***Obituary.***

James Copland, M.D., F. R. S., the well known author and eminent physician, died in London on the 12th July last, of an attack of hæmaturia connected with prostatic disease followed by uræmia, æt. 78. He graduated in 1815. His "Encyclopædic Dictionary of the Medical Sciences," which occupied thirty years of his life, is everywhere known and highly appreciated. Every line of it was written by his own hand, and he personally revised and corrected the proof-sheets. Probably no one man since John Mason Good has accomplished so much by his own unaided industry.——Landon C. Rives, M.D., who died recently in Cincinnati, at the age of 80, was formerly associated in the faculty of Cincinnati College with Drs. Drake, Gross, Parker, Rogers, Harrison, and McDowell, certainly one of the ablest associations of Medical teachers ever known in this country. Drs. Gross and Parker are now the only survivors.——Dr. Auzias Turenne, the syphilographer and experimenter on syphilization, died in Paris, May 29th, aged 59.——Sir James Clark, known as Physician in Ordinary to Queen Victoria, and for his works on climate and consumption, died June 29, at the age of 81.——Gunning S. Bedford, M.D., Professor in the University Medical College of New York, author of several works popular with the profession, died in New York on the 5th ult.

